# Association of Mammographic Density with Pathologic Findings

**DOI:** 10.5812/ircmj.16698

**Published:** 2013-12-05

**Authors:** Nasrin Ahmadinejad, Samaneh Movahedinia, Sajjadeh Movahedinia, Mona Shahriari

**Affiliations:** 1 Advanced Diagnostic and Interventional Radiology Research Center (ADIR), Tehran University of Medical Sciences (TUMS), Tehran, IR Iran; 2 School of Medicine, Tehran University of Medical Sciences (TUMS), Tehran, IR Iran

**Keywords:** Mammographic Density, Pathology, Receptors, Estrogen, Receptors, Progesterone

## Abstract

**Background:**

Breast cancer is one of the most common cancers in the world and is the first cause of death due to cancer among women. Mammography is the best screening method and mammographic density, which determines the percentage of fibro glandular tissue of breast, is one of the strongest risk factors of breast cancer. Because benign and malignant lesions may present as dense lesions in mammography so it is necessary to take a core biopsy of any suspicious lesions to evaluate pathologic findings.

**Objectives:**

The aim of this study was to assess the association between mammographic density and histopathological findings in Iranian population. Moreover, we assessed the correlation between mammographic density and protein expression profile. We indeed, determined the accuracy and positive predictive value and negative predictive value of mammographic reports in our center.

**Patients and Method:**

This study is a cross-sectional study carried out among 131 eligible women who had referred to imaging center for mammographic examination and had been advised to take biopsy of breast tissue. All participants of the study had filled out the informed consent. Pathologic review was performed blinded to the density status. Patients were divided into low density breast tissue group (ACR density group 1-2) and high density breast tissue group (ACR 3, 4) and data was compared between these two groups. Statistical analysis performed using SPSS for windows, version 11.5. We used chi-square, t-test, and logistic regression test for analysis and Odds Ratio calculated where indicated.

**Results:**

In patients with high breast densities, malignant cases (61.2%) were significantly more in comparison to patients with low breast densities (37.3%) (P= 0.007, OR=2.66 95% CI=1.29-5.49). After adjusting for age, density was associated with malignancy in age groups <46 years (P=0.007), and 46-60 years (P=0.024) but not in age group >60yrs (P=0.559). Adjusting for menopausal status, density showed association with malignancy in both pre-menopause (P=0.041) and menopause (P=0.010) patients. Using logistic regression test, only age and density showed independent association with risk of breast cancer. No association was found between density and protein profile expression. Mammographic method has a false negative percent of 10.3% for negative BI-RADS group and a Positive Predictive Value (PPV) of 69.6% for positive BI-RADS group. PPVs for BI-RADS 4a, 4b, 4c and 5 were 16%, 87.5%, 84.6%, and 91.5% respectively. NPVs for BI-RADS 1, 2 and 3 were 66.7%, 95.8% and 90.0% respectively.

**Conclusions:**

In this study we found that increasing in mammographic density is associated with an increase in malignant pathology reports. Expression of ER, PR and HER-2 receptors didn't show association with density. Our mammographic reports had a sensitivity of 94.1% and a specificity of 55.6%, which shows that our mammography is an acceptable method for screening breast cancer in this center.

## 1. Backgrtound

Breast cancer is one of the most common cancers in the world and is the first cause of death due to cancer among women (1, 2). In Iran, breast cancer accounts for about 24.4% of all neoplasms among women (3).

Breast cancer is correlated with several genetic and environmental factors such as mutations in BRCA1 and BRCA2 (4, 5), estrogen expression and mammographic density, as the strongest ones (6, 7).

Mammography is the best screening method, which can help us diagnosis breast cancer in asymptomatic stages. Mammographic density determines the proportion of fibro glandular area to total breast area in mammographic images. Based on BI-RADS (Imaging-Reporting and Data System) classification, mammographic density has been categorized into four groups, (I) almost fat; (II) scattered fibro glandular densities, (III) heterogeneously dense, (IV) extremely dense (7).

As the radiological appearance of breast lesions are similar to fibro glandular breast tissue, many common (8, 9) and uncommon (10, 11) benign and malignant lesions may present as dense lesions in mammography that affect mammographic density sensitivity in predicting risk of malignancy. One limitation of automated methods in estimating underlying breast density is that such lesions are not ignored and semi-automated methods, in which the radiologist specifies lesions, seem more precise. On the other hand, in case of dense breasts, the breast tissue density may obscure underlying lesions. Thus it is necessary to take a core biopsy of any suspicious mammographic or clinical finding when density is high (12, 13). Based on BIRADS classification, women who have suspicious mammographic BIRADS 4 and 5, should be examined microscopically.

As mentioned above, high mammographic density increases the risk of breast cancer. To our knowledge, there is no study investigating on this association in Iranian population. On the other hand, the correlation between mammographic density and protein expression profile (ER/PR/HER-2) is not clear.

## 2. Objectives

The aim of this study was to assess the association between mammographic density and malignant histopathological findings, moreover the correlation between mammographic density and protein expression profile. Meanwhile, we evaluated the correlation of mammographic reports and pathologic results to determine the accuracy and positive predictive value and negative predictive value of mammographic reports.

## 3. Patients and Methods

This study is a part of another analytical cross-sectional study carried out in Tehran, Iran to evaluate breast density distribution among Iranian population and assess its association with breast cancer risk factors. Sampling continued to achieve enough eligible biopsied cases according to the present study estimated sample size. 

Participants were among women who had referred to imaging center of Imam Khomeini Cancer Institute for mammographic examination, all reported by the same radiologist and advised to take biopsy of breast tissue. Biopsied cases included individuals with suspicious findings in mammography (BI-RADS categories 4 and 5), those with suspicious findings in subsequent imaging work up (mammographic initial BI-RADS category 0), and those who are advised to undergo biopsy because of dense breast tissue or clinically suspicious findings despite benign findings in mammograms. Biopsy was a part of the diagnostic process in all patients and tendency or dissatisfactory in participating in the study, did not affect their diagnostic or therapeutic outcomes. All participants of the study had filled out the informed consent and considering the need to follow up patients until getting pathology results, they could quit the study any time during this period. According to previous studies in our center, the radiologists had not enough inter-observer agreement in reporting the BI-RADS category (kappa=0.300), which determines who to be biopsied. Therefore, it was necessary to select cases reported by one observer. Inclusion criteria: Accessibility to mammographic report, including mammographic density and BI-RADS category, reported by the radiologist participating in the study and accessibility to pathology report.

Exclusion criteria: history of bilateral breast cancer or bilateral mastectomy, any personal history of breast cancer in benign pathology reports, history of breast cancer or breast surgery or radiotherapy on the same breast that had been biopsied in malignant cases. In malignant cases with a history of contra lateral breast cancer, the mammographic information of the contra lateral breast was included in the analysis.

All patients filled out the demographic questionnaire asking about age, menopausal status, etc. An expert breast radiologist, using BI-RDAS standard lexicon, reported mammograms. All mammograms (full-digital two-view ones) were taken with the same technique and read on the same system. Pathologic review performed blinded to the density status.

Patients were divided into low density breast tissue group (ACR 1-3) and high density breast tissue group (ACR 4, 5) and Pathological findings were compared between these two groups as well as the correlation between mammographic density with histopathological findings and protein expression profile (ER/PR/HER-2). We also evaluated the association between malignant or benign pathological findings with positive (BIRADS 4, 5) or negative (BIRADS 1-3) mammographic reports. Positive and negative predictive value for mammographic reports was determined.

Statistical analysis performed using SPSS for windows, version 11.5. We used Qi square, t-test, and logistic regression test for analysis and Odds Ratio calculated where indicated.

## 4. Results

131 eligible women, who had been undertaken breast biopsy, were entered into the study. Participants included 63 women (48.1%) with benign pathology reports, 60 patients (45.8%) with invasive breast carcinoma and 8 women (6.1%) with in situ carcinoma. 

Participants mean age was 47.9 (SD= 10.5). 41.9% of participants were menopause.

Patients’ percentage in the four mammographic density categories was 9.9%, 30.5%, 43.5% and 16% respectively. 70.2% of biopsied cases had a positive mammographic BIRADS (4 and 5) and 29.8% of them had a negative one (BIRADS 1-3). In the first group high-density breasts were 66.3% versus 48.7% in the second group without any statistically significant difference (P=0.059).

In patients with high breast densities, malignant cases (61.2%) were significantly more in comparison to patients with low breast densities (37.3%) (P= 0.007, OR=2.66 95% CI=1.29-5.49) (Table 1).

Results of t-test showed that mean density in benign cases (49.4%) is significantly less than that of malignant ones (58.1%) (P=0.022 Mean Difference=8.7 95% CI=1.26-16.11).

**Table 1.** Association of Density and Histopathologic Characteristics and other Associated Factors

Variables	High Density (n=77) No. (%)	Low Density (n= 47) No. (%)	P-value OR (95% CI)
**Pathology**			0.007^a^, 2.66 (1.29-5.49)
Benign	31(38.8)	32(62.7)	
Malignant	49(61.2)	19(37.3)	
**ER**			0.602^b^
Positive	24 (82.8)	5 (71.4)	
Negative	5 (17.2)	2 (28.6)	
**PR**			0.686^b^
Positive	19 (65.5)	4 (57.1)	
Negative	10 (34.5)	3 (42.9)	
**HER-2**			1.000^b^
Positive	10 (38.5)	3 (42.9)	
Negative	16 (61.5)	4 (57.1)	
**Menopausal status**			<0.001^a^, 0.26 (0.12-0.57)
Yes	23 (29.9)	29 (61.7)	
No	54 (70.1)	18 (38.3)	

^a^Derived from Chi-square test

^b^Derived from Fisher Exact test

Mean age of women in benign group is higher than malignant one (46.2 vs. 49.7) with near significant difference (P= 0.064). After adjusting for age, the association still existed in age groups <46 years (P=0.007), and 46-60 years (P=0.024) but not in age group >60yrs (P=0.559). Adjusting for menopausal status, density showed association with malignancy in both pre-menopause (P=0.041) and menopause (P=0.010) patients.

Using logistic regression test, interaction of all these factors was evaluated which showed that only age and density were associated with risk of breast cancer (Table 2). 

**Table 2.** Results Obtained from Logistic Regression Analysis for Malignancy

	Negative BIRADS (n=39)		Positive BIRADS (n=92)		Total (n=131)	
	P-value, Unadjusted OR (95%CI)	P-value, Adjusted OR (95%CI)	p-value, Unadjusted OR (95%CI)	P-value, Adjusted OR (95%CI)	P-value, Unadjusted OR (95%CI)	P-value, Adjusted OR (95%CI)
**Age**	0.046, 0.13 (1.002-1.28)	0.075, 1.17 (0.98-1.40)	0.524, 1.01(0.97-1.06)	0.323, 1.04 (0.96-1.13)	0.008, 2.66 (1.29-5.49	0.010, 1.09 (1.02-1.16)
**Menopausal status**	0.212	0.708	0.736	0.884	0.565	0.304
**Menopaused, Non menopaused**	4.50 (0.42-47.76)	0.47 (0.01-23.77)	1.18 (0.46-23.77)	1.12 (0.24-5.29)	1.23 (0.60-2.52)	0.53 (0.16-1.77)
	1 (ref)	1 (ref)	1 (ref)	1 (ref)	1 (ref)	1 (ref)
**Density percent**	0.642, 1.01 (0.96-1.06)	0.309, 1.03 (0.97-1.10)	0.031, 1.02 (1.002-1.05)	0.012, 1.03 (1.01-1.06)	0.024, 0.02 (1.003-1.037)	0.002, 1.03 (1.01-1.05)

ER positive breast cancers (82.8%) were more in patients with dense breasts compared to patients with low-density breasts(71.4%) but this difference was not statistically significant (p=0.602). PR positive breast cancers were also more in patients with dense breasts (65.5% vs. 57.1%) without any significant difference (P=0.686).

Patients with dense breasts showed less HER-2 positive cancers (38.5%) compared to low density group (42.9%) which showed no significant difference (P=1.000).

T-test revealed that mean breast density was more in ER-, PR+ and HER-2+ groups, all without any significance (Table 1). 

One-way ANOVA showed no significant difference in mean breast density of different histological grades (P=0.671).

Among 39 patients with negative BI-RADS reported in mammography, four patients’ pathology report was demonstrative of malignancy, which shows a Negative Predictive Value (NPV) of 89.7% or a false negative percent of 10.3% for this screening method. Among 92 patients with positive mammographic BI-RADS, 64 individuals showed malignancy in pathologic examination introducing a Positive Predictive Value (PPV) of 69.6%.According to above findings our mammographic reports had a sensitivity of 94.1% and a specificity of 55.6%.

PPVs for BI-RADS 4a, 4b, 4c and 5 were 16%, 87.5%, 84.6%, and 91.5% respectively. NPVs for BI-RADS 1, 2 and 3 were 66.7%, 95.8% and 90.0% respectively. 

## 4. Discussion

In this study we found that increasing in mammographic density is associated with an increase in malignant pathology reports (P= 0.007). Expression of ER, PR and HER-2 receptors did not show any association with density. PPVs for BI-RADS 4a, 4b, 4c and 5 were 16%, 87.5%, 84.6%, and 91.5% respectively. Our mammographic reports had a sensitivity of 94.1% and a specificity of 55.6%, which shows that it is an acceptable method for screening breast cancer. 

For individual-dependent imaging methods, studying their association with more objective related factors (like association of BIRADS with pathology, strong risk factors of breast cancer or breast density) helps to evaluate such methods, but without defining the final BIRADS of zero ones who have mostly dense breasts, we cannot get reliable results. In centers with poor follow up of patients, it is better to decrease the recall rate as much as it is possible. The best way for evaluating this screening way is to assess the association between BIRADS and subsequent pathology results.

It has been established in previous studies that breast density is associated with invasive (14, 15) and in situ breast cancer (14, 16) which is similar to our findings. It has been recognized that abnormal stromal and epithelial environment increases the risk of malignancy (17) and density is positively associated with histologic grade and mitotic index (18) so increased epithelial cell proliferation due to increased density may be an explanation for association between breast density and malignancy. However, in our study density was not associated with grade, despite its association with malignancy.

In our study in the positive mammographic BIRADS categories (4 and 5), high-density breasts were 66.3% versus 48.7% in the negative ones (BIRADS 1-3); Considering the fact that some of the patients with probably benign breast lesions are recommended to undergo biopsy due to their dense breast tissue, which may obscure underlying detail, this finding is explainable. This selection bias negatively confounds the association between density and risk of malignancy, so in case of finding an association between mammographic density and breast cancer, the association is expected to be stronger than what analysis shows.

We get results that are more precise, if we compare mean breast density percent in malignant and benign groups. The advantage of this method is that the difference between each of four density categories is taken into consideration and the difference between ACR categories II and III is not that exaggerated as the difference between categories I and IV. The results showed significant association between breast density and malignancy as we expected.

Mean age of women in benign group was higher than malignant one with near significant difference (P= 0.064) proposing that age and menopausal status may play a role as confounders in the association above perhaps because of their effects on Breast density (19, 20). Adjusting for age and menopausal status, the association still existed between density and risk of malignancy.

In this study, we found no significant association between breast density and expression of ER, PR and HER-2 receptors. The association between these receptors and breast density has been investigated in several studies. Some of them found no association which was similar to our findings (18, 21) with the exception in low grade symptomatic women (21). Roubidoux et al. (22) discussed that increasing density is associated with ER negatively cancers and poor prognosis (22). Another study showed that density is a risk factor for both ER positive and ER negative tumors (23). These findings support the hypothesis that breast density and estrogen expression affects breast cancer through independent pathways (23, 24).

As a screening method, mammography should have a high sensitivity and NPV not to miss patients; however, it would better to have an acceptable specificity to save costs and prevent unnecessary biopsies. Mammography sensitivity is dependent on the quality of equipment, competence of radiology staff and the density of the breast tissue (25), so high-density areas in mammogram may mask breast lesions and reduce the chance of early detection of cancerous lesions (26). Our mammographic reports had a sensitivity of 94.1% and a specificity of 55.6% which it has high and acceptable sensitivity in comparison with other studies (27-29). Choi et al. (12) conducted a study on the accuracy of BI-RADS and concluded that mammographic BI-RADS had a sensitivity of 78% and a specificity of 57% (30). Another study carried out by Filho et al. (24) proposed a sensitivity of 93.3%, a specificity of 95%, a PPV of 75.2% and a NPV of 98.8% for mammographic BI-RADS, introducing this method as an accurate way of defining the nature of breast lesions (30).

Since probability of malignancy differs between different mammographic BI-RADS categories, it would be better to report PPVs and NPVs separately for each one to have better assessment of our mammographic reports. PPVs for BI-RADS 4a, 4b, 4c and 5 were 16%, 87.5%, 84.6%, and 91.5% respectively which was nearly similar to other studies specially for BI-RADS 5 (31-35). Standard PPV defined for BI-RADS 5 is percents more than 95%.

Despite what expected, NPV for BI-RADS 1 is not acceptable, but considering that only three patients were among this category, this finding is not that reliable for judgment. However, the one patient with pathology-mammography discordance in this category had extremely dense breast tissue, and was advised to undergo biopsy. This emphasizes the importance of considering density in management of mammograms.

Our study was limited because of absence of regular mammographic screening programs in Iran, thus it was impossible to design a retrospective study, which has intervals between mammographic screening and cancer diagnosis.

Based on our findings mammography is the best method for early detection of breast cancer in asymptomatic patients and has high positive and negative predictive values. It seems to be necessary to arrange screening programs in Iranian population, who has a higher prevalence of breast cancer in younger ages. Indeed, mammography should be performed at regular intervals for individuals who have high breast density in order to make early diagnosis in those who developed a suspicious lesion in routine screening.
